# Crossbreeding and Backcrossing in the Pyrethroid-Resistant Ladybird Beetle *Eriopis connexa* (Germar) Determines Resistance in Offspring

**DOI:** 10.3390/insects15110853

**Published:** 2024-10-31

**Authors:** Alice S. Rodrigues, Paulo R. R. Barbosa, Deividy V. Nascimento, Jorge B. Torres

**Affiliations:** 1 DEPA-Entomologia, Universidade Federal Rural do Pernambuco (UFRPE), Rua Dom Manoel Medeiros s/n, Dois Irmão, Recife 52171-900, PE, Brazil; alicesutanarodrigues@gmail.com (A.S.R.); deividysx@gmail.com (D.V.N.); 2 Instituto de Ciências Agrárias, Universidade Federal dos Vales do Jequitinhonha e Mucuri, Campus Unaí, Av. Universitária, n. 1000, Unaí 38610-000, MG, Brazil; paulo.barbosa@ufvjm.edu.br

**Keywords:** resistance to pyrethroids, offspring performance, resistance stability, conservation, biological control

## Abstract

The conservation of the predatory ladybird beetle, *Eriopis connexa* (Germar) after its release also relies on its resistance and the performance of its progenies. When resistant individuals are released or evolve in the field through mating with susceptible pairs, we anticipate that they will generate a resistant progeny, inheriting the resistance to lambda-cyhalothrin through an autosomal mechanism. The susceptibility of a field-collected population (EcGA) was characterized and determined by the performance of their progenies generated through mating with a resistant parent (EcViR). We paired virgin adults from the EcGA and EcViR populations, observing how their progenies developed, reproduced, and survived when exposed to dried lambda-cyhalothrin residues applied at field rates. Adults from the EcGA population were ~200-fold more susceptible than those of the EcViR population. Developmental times from larva to adult emergence for EcGA individuals were delayed by approximately 6 days and generated smaller adults compared to EcViR and their progenies. The egg production did not differ across parents and progenies, but females from EcGA and EcGA × EcViR progenies produced 130 more eggs in comparison to EcViR females during the 35-day evaluation period. Exposure to lambda-cyhalothrin resulted in 77.4 to 100% survival for adults from EcViR × EcGA progenies and EcViR parents, while EcGA and the population standard for susceptibility maintained in the laboratory (EcFM) did not survive the insecticide exposure. These findings indicate that field crosses between EcViR and EcGA will improve their progenies’ biological performance compared to the EcViR parents and will maintain a high lambda-cyhalothrin survival rate.

## Simple Summary

Insecticides and augmentative biological control employing the inundative release of natural enemies are strategies to obtain a rapid reduction in pest populations. Regardless of insecticide selectivity, the dominant outcome is the incompatibility between these two control approaches. The majority of natural enemies are also susceptible to insecticides, and a physiologically selective insecticide may feasibly integrate both methods. Resistant natural enemies are those that were once susceptible but have acquired physiological changes that confer resistance to insecticides analogous to pest species under insecticide pressure. As a result, natural enemies that are selected for resistance to common nonselective insecticides fall under the category of physiological selectivity. Insecticides from the pyrethroids group are primarily recommended for managing defoliating insect pest species, whereas the ladybird beetle Coccinellinae predominantly preys on aphids. This means that they control distinct pest categories in the agroecosystem. Reports also state that pyrethroid treatments cause outbreaks of sucking insect pests like aphids, whiteflies, and psyllids because they negatively impact their natural enemies, with a small impact on the sucking insect pest species. The presence of pyrethroid-resistant ladybird beetles is essential for integrating these two control methods.

## 1. Introduction

A species’ maintenance in the environment depends on its fitness, which is defined as the ability of the offspring to survive and successfully reproduce. To a greater or lesser extent, synthetic insecticides are a stressor in crop ecosystems for arthropod fitness, particularly for natural enemies that are susceptible to insecticides [1]. Although broad-spectrum insecticides are used to control pest species, they can also have an impact on natural enemies’ survival, reproduction, development, and behavior [2]. Lady beetles do not attack lepidopteran and coleopteran pests that are targeted by broad-spectrum insecticides such as pyrethroids [3]. Otherwise, because of their broad-spectrum mode of action, pyrethroids have often correlated to outbreaks of sap-sucking pest species such as aphids due to their negative impact on aphidophagous species [4,5,6,7,8]. Despite their non-target impact, pyrethroids are still widely used due to their relatively low cost, broad market availability, low toxicity to mammals, and quick knockdown effect [9,10].

The reduction in ladybird beetle populations in crop systems treated with broad-spectrum insecticides can be related to either the insecticide’s toxicity or the removal of preferential prey items after the insecticide treatment [11,12,13]. However, it is important to note that the successive exposure of pest or predator species to the same insecticide can select resistant populations of pest species as well as ladybird beetles [14,15,16,17], which may have an impact on integrated pest management directly or indirectly.

Regarding insects, biology (e.g., the type of reproduction and voltinism [18,19]), physiology (e.g., metabolic activity [20]), and even behavior (e.g., avoidance of treated areas [21]) are traits that can accelerate or slow down the evolution of resistance in a population. Characteristics inherent to resistance include the mechanism by which the insect becomes insensitive to the toxic compound and its mode of inheritance, both of which influence its evolution. Despite records of target site alteration [22,23,24], metabolic detoxification is considered a mechanism associated with insect resistance to pyrethroids [14,20,25], as demonstrated by *Eriopis connexa* (Germar) (Coleoptera: Coccinellidae). Furthermore, resistance to pyrethroids has also been characterized as autosomal, ranging from incompletely dominant to incompletely recessive [20,26,27]. However, variations can occur in relation to sex linkage [28,29] and the number of associated genes [27,30,31]. Collectively, this information explains, at least in part, the large number of cases of insect resistance to pyrethroids (https://pesticideresistance.org/, accessed on 13 January 2021) in various agroecosystems.

Like arthropod pest species, natural enemies are also subject to selection for insecticide resistance in the field. The predator *Chrysoperla carnea* (Stephens) (Neuroptera: Chrysopidae) developed resistance to α-cypermethrin, cypermethrin, deltamethrin, fenvalerate, lambda-cyhalothrin, and permethrin [14,15,32], while *Chrysoperla externa* Hagen (Neuroptera: Chrysopidae) is reported to be resistant to lambda-cyhalothrin [33,34]. Among ladybird beetle species (Coleoptera: Coccinellidae), *E. connexa* has been characterized as resistant to lambda-cyhalothrin [16], and with high tolerance to several other pyrethroids [35]; *Hippodamia convergens* Guérin-Méneville is resistant to lambda-cyhalothrin [20], to lambda-cyhalothrin, and to dicrotophos [36]; *Stethorus gilvifrons* Mulsant to bifenthrin [37]; *Propylaea japonica* (Thunberg) to imidacloprid [38] and to chlorpyrifos, methamidophos, fenvalerate, and to avermectins [39]; and *Coccinella septempunctata* L. has been selected for resistance to thiamethoxam [40].

Resistance to insecticides in natural enemy populations opens up a range of opportunities by enabling the integration of insecticides and biological control methods, as pursued within IPM by the physiological selectivity of insecticides [1,41,42]. This is because resistant natural enemy populations can withstand field applications of previously non-selective insecticides [1,43]. The ladybird beetle *E. connexa*, the natural enemy species studied herein, is a lambda-cyhalothrin-resistant population. The resistance of *E. connexa* was revealed to be autosomal and partially dominant [20,44]. This resistance is achieved through metabolic detoxification via the enhanced activity of alpha and beta esterases [20]. Following this characterization, research has revealed that females in the resistant population have 50% lower fecundity [45,46] and survival [47] than females in the susceptible population. However, crossing resistant and susceptible beetles resulted in offspring with greater fecundity and survival [47,48]. In the absence of selection pressure, resistance remained stable at 50% of its initial resistance ratio [42], and reselection of F1 revealed a return of the reproductive cost [47]. Moreover, when resistant individuals are crossed with susceptible ones, the data indicate that the resistance ratio diminishes significantly after three generations of crossing in the absence of any selection pressure [38,47].

According to these findings, pairing resistant with susceptible beetles results in an F1 generation that can tolerate lambda-cyhalothrin treatment and generate more offspring than its parentals. Lambda-cyhalothrin, a broad-spectrum pyrethroid, is indicated for use against a variety of pest species. Brazil currently provides 38 insecticide formulations using lambda-cyhalothrin, either as a single ingredient or in a ready-to-use formulation with diamides, sulfoximines, and neonicotinoids [49]. Despite its ability to control a wide range of pest species, lambda-cyhalothrin in a single formulation has minimal efficiency against sucking insect pests. In reality, its treatment affects natural enemies, resulting in aphid outbreaks. Aphidophagous species like *E. connexa* may help control aphids when pyrethroids like lambda-cyhalothrin are applied against other target pest species. This study tested the hypothesis that the lambda-cyhalothrin-resistant population of *E. connexa*, under selection pressure in the laboratory, performs better when subjected to reciprocal crosses and backcrosses with resistant individuals, simulating the incipient population produced in the field after releasing resistant individuals. The findings not only aid in understanding the performance of F1 individuals obtained after releasing resistant ones, but also provide guidance on how to increase offspring production when rearing resistant populations in the laboratory. This can be achieved by crossing and backcrossing populations without losing the resistant trait.

## 2. Materials and Methods

### 2.1. Ladybird Beetle Populations

Three populations of *E. connexa* were reared for this study in an acclimatized room (25 ± 1 °C, relative humidity 60–70%, and 12:12 h (L:D) photoperiod). One population susceptible to lambda-cyhalothrin was newly established from individuals collected in soybean remains and chickpea located in “Núcleo Rural Vargem da Bênção, BR 060 Km9, Gama, DF (−15.9166, −48.13811) (hereafter termed the ‘EcGA’ field population). Two other populations were reared: one resistant to lambda-cyhalothrin [16,20], which was reared for 78 generations under lambda-cyhalothrin selection pressure (EcViR), and one susceptible population (EcFM) reared for 76 generations without insecticide exposure.

Adults of each of three ladybird beetle populations were reared separately using Plexiglas^®^ cages (Macril Artes em Acrílico, Recife, PE, Brazil) (60 × 30 × 30 cm in L × W × Ht) containing lateral openings fixed with organdie fabric for ventilation. Eggs of *Ephestia kuehniella* (Zeller) (Lepidoptera: Pyralidae) plus a paste made of honey–yeast 1:1 were provided ad libitum as food. Wrinkled towel paper was offered as oviposition substratum. Egg batches were collected daily and incubated in plastic pots of 80 mL until hatching. Hatched larvae were reared at a rate of three larvae per 80 mL pot and fed *A. kuehniella* eggs replaced every other day up to the metamorphose for pupa. Adults were used in the experiments or used to collect eggs, starting a new generation of each population.

### 2.2. Lambda-Cyhalothrin

The experiments used both technical-grade and commercial formulations of lambda-cyhalothrin. For the dose–mortality tests, we used technical grade (99.5%, Chem Service, West Chester, PA, USA) lambda-cyhalothrin that had been diluted in acetone PA-ACS (99.5%, Bioquímica e Química Ltd.a, Belo Horizonte, MG, Brazil). In the residual bioassays, we diluted lambda-cyhalothrin at the label dose in the commercial formulation Karate Zeon^®^ 50SC (lambda-cyhalotrin 5% *w*/*v*—50 g L^−1^, Syngenta S.A., São Paulo, SP, Brazil) at 0.1% aqueous surfactant solution (Wil-Fix, Charmon Destyl Industrial Qumica Ltd.a, Campinas, SP, Brazil) as a control.

### 2.3. Dose–Mortality Bioassays

The bioassays were conducted using lambda-cyhalothrin at technical grade to determine the susceptibility of the field-collected (EcGA) population and the response of the two lab-reared populations, EcViR and EcFM. Adult beetles 8–10 days old received 0.5 mL of insecticide diluted in acetone applied on the abdominal venter using a 25 µL Hamilton™ syringe (Hamilton Company, Reno, NV, USA). Preliminary bioassays indicated the doses required to obtain mortality greater than 0% and lower than 100%. Therefore, we applied six doses ranging from 0.001 to 0.05, 0.05 to 0.35, and 1.0 to 10.0 mg a.i. mL^−1^ to the adults of the EcGA, EcFM, and EcViR populations, respectively. Each dose was assessed against a minimum of 20 beetles without separating by gender. After the treatment, the insects were placed in Petri dishes (10 cm diameter × 1.5 cm height) lined with filter paper and fed with *A. kuehniella* eggs. Mortality was assessed 48 h after adult treatment.

### 2.4. Biological Characteristics of Susceptible and Resistant Crossbreed of Eriopis connexa

Previous studies found a reduction in fecundity of lambda-cyhalothrin-resistant females (EcViR) [45,46]. Thus, a series of studies were conducted crossing the recently field-collected and susceptible population (EcGA) and the resistant population EcViR, followed by backcrossing the F1 crossbreed with EcViR. Males and females were separated at the emergence date to guarantee their virgin status. We treated adult EcViR 5-day-olds with 6.0 mg a.i. mL^−1^ to identify only homozygous resistant individuals for crossbreeding with susceptible individuals. Further, one parental EcViR was singly paired with a susceptible male or female to certify that only an EcViR parent crossed with a parent of the designed cross (Table 1). Three crosses were established: three referential crosses with parents from the same population (i), two reciprocal crosses using EcGA and EcViR parents (ii), and the backcrosses of F1 with reciprocal crosses (ii) with EcViR (iii). Each cross was run with 15 pairs (replications) using unmated adult beetles 7–10 days old, except the referential crosses EcFM and EcViR that had only 10 replications. The pairs were reared in 80 mL pots containing pieces of paper towel as substratum for oviposition. The larvae hatched from the second and third egg batches were used to assess offspring performance. Sixty larvae were monitored to determine pre-adult development, viability, and the fresh body mass of the emerged adults. Adults were paired when 5 days old and daily inspected for egg deposition and female survival for 35 days.

### 2.5. Crossbreed Offspring Response to Label Doses of Lambda-Cyhalothrin

The survival of offspring adults exposed to lambda-cyhalothrin was determined for reciprocal cross and backcross beetles. Adult beetles were exposed to lambda-cyhalothrin dry residues obtained with the lowest and highest label doses of Karate Zeon^®^ 50SC (100 and 400 mL per hectare) recommended against cotton pests [49]. These label doses correspond to 5 and 20 g of a.i. per hectare (ca. 0.033 and 0.133 mg a.i. mL^−1^ using a 150 L water spraying volume).

Both the bottom and top of glass the Petri dishes, measuring approximately 9 cm in diameter and 1.2 cm in height, were treated with 1 mL of either the control or insecticide solution. The solution was left to air-dry for about 2 h inside a Nalgon 3700 exhaust chamber (Nalgon Equipamentos Científicos, Itupeva, São Paulo, Brazil) before placing the test beetles inside the dishes. Eggs of *E. kuhniella* were provided ad libitum as food inside the dishes. Seven treatments were established, represented by offspring from the reference, reciprocal crosses, and backcrosses with three replications (dishes) each. Depending on availability, we confined eleven to 25 adult beetles per dish, resulting in an evaluation of 40 to 75 beetles per treatment. The knockdown effect and mortality were recorded 2 h and 48 h after placing the beetles inside the dishes.

### 2.6. Statistical Analysis

The susceptibility of adult beetles from three *E. connexa* populations to lambda-cyhalothrin was investigated through the lethal dose calculation. The PROC PROBIT of SAS [50] was used to calculate the lethal doses (LDs) of lambda-cyhalothrin and their respective 95% fiducial limits. For the comparisons, the population with the lowest LD50 (EcGA) was used as the standard of susceptibility. Furthermore, we estimated the resistance ratio (RR) and its respective 95% confidence intervals (CIs), characterizing significance when the CI did not include the value 1.0 [51,52].

We checked the developmental times, immature viability, adult fresh body weight, adult fecundity, and percentage of survival data for normality (Shapiro–Wilk’s test, PROC UNIVARIATE) and homoscedasticity (Lavene’s test, PROC ANOVA) and transformed them as necessary to fit these ANOVA assumptions. We performed one-way ANOVA and Tukey’s HSD approaches to analyze and compare the data, with the exception of the survival data. The percentage of survival was submitted to a two-way ANOVA considering a 7 × 2 design represented by seven treatments and two lambda-cyhalothrin label doses. Survival curves were calculated by the Kaplan–Meier method, and their comparisons were performed through a log-rank test using the PROC LIFETEST of SAS [50].

## 3. Results

### 3.1. Dose–Mortality Bioassays

The mortality data for the three *E. connexa* populations tested fit the Probit model (*p* > 0.05), allowing for inferences concerning the calculated LD_50_. The EcViR population had the highest LD_50_, followed by the EcFM population and the EcGA field population (Table 2). Thus, the EcGA field population was used as a standard of susceptibility for comparison with the others, yielding a statistically significant RR50 compared to the EcViR population (Table 2).

### 3.2. Biological Characteristics of Susceptible and Resistant Crossbreeds of Eriopis connexa

The egg production per female of HA and HB backcrossed with EcFM males was similar to that of EcViR and EcGA females (reciprocal crosses) and EcFM (reference cross), but significantly higher than that of EcViR females (reference cross), with EcGA females (reference cross) producing an intermediate number of eggs (Table 3). Furthermore, eggs from HA and HB were more viable than those from the other crosses, although EcGA females crossed with EcViR males deposited the least viable eggs. The EcGA larvae required about one week longer to complete their development compared to the other crosses, which did not differ from each other. Additionally, the pupal period of HB was significantly longer than that of EcFM, followed by EcGA, HA, and EcViR, with no difference between the last two groups. The descendants of the reference and reciprocal crosses (EcGA, EcFM, EcViR, HA, and HB) did not differ in terms of egg-to-adult survival rate or fresh weight for newly emerging adults.

### 3.3. Crossbreed Offspring Response to Label Doses of Lambda-Cyhalothrin

**Figure 1:**
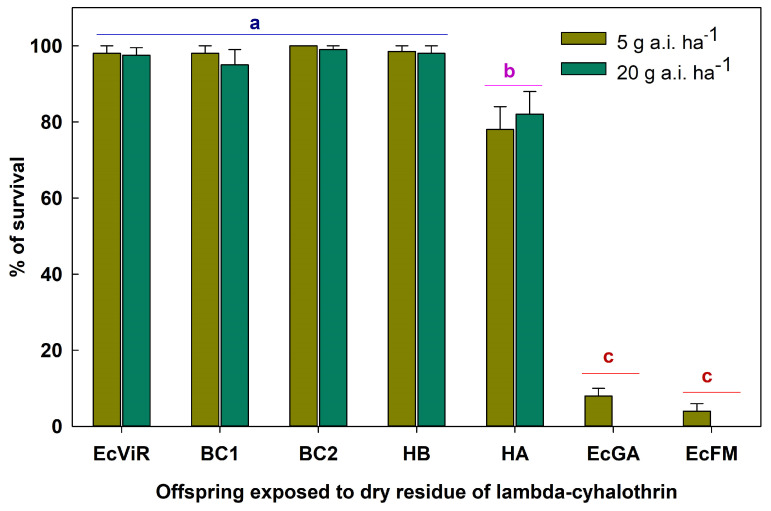
Survival of *Eriopis connexa* adults from resistant (Ec-ViR) and susceptible reference populations (Ec-FM and Ec-GA), backcrossed progeny (BC1 and BC2), and reciprocal crosses (HB and HA), assessed on dry residues on inert surfaces treated with the lowest and highest label dose of lambda-cyhalothrin. See Table 1 for more details. Note: bars with different letters indicate a significant difference (Tukey HSD’s test, a = 0.05).

There was no mortality in the control groups that had no contact with lambda-cyhalothrin residues. As a result, the survival rate of insects exposed to lambda-cyhalothrin residue for 48 h at both the lowest and highest recommended field doses did not need to be corrected for natural mortality. The exposure to insecticide residues had no effect on the survival of offspring from the various established crossings (F = 0.39; df = 1, 40; *p* = 0.5370). Similarly, there was no interaction effect between the recommended doses of lambda-cyhalothrin and the offspring from different crossings (F = 0.62; df = 6, 40; *p* = 0.7133). Meanwhile, the survival rate of EcViR, HA, HB, BC1, and BC2 offspring was significantly higher than that of the EcGA and EcFM offspring, which did not differ from each other (F = 539.13; df = 6, 40; *p* < 0.0001) (Figure 1).

**Figure 2:**
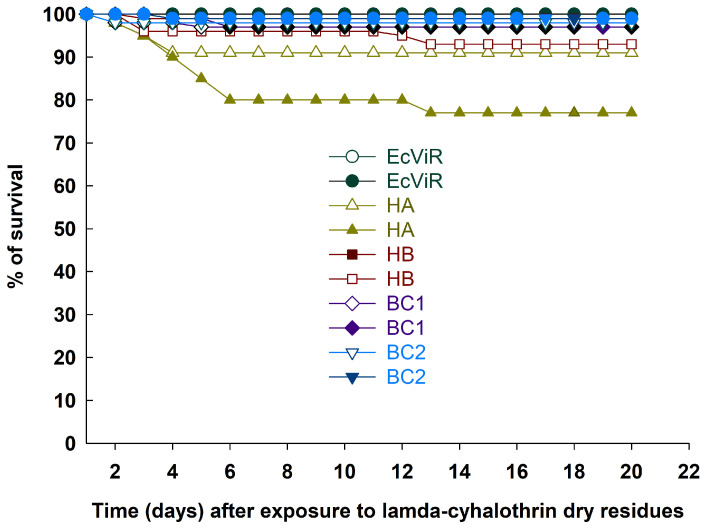
Survival curves of adult *Eriopis connexa* from different parental populations, crossed, and backcrossed offspring (see Table 1), during 20 days after recovering from exposure to the dry residue of lambda-cyhalothrin (Karate Zeon 50CS) at the lowest (5 g a.i. ha^−1^ open symbols) and highest (20 g a.i. ha^−1^ closed symbols) recommended label doses against cotton pests in Brazil. Note: curves estimated by the Kaplan–Meier method and compared using the log-rank test (α = 0.05); some curves and symbols overlap in value.

The insects that survived exposure to lambda-cyhalothrin residue died differently over 20 days (Log-rank test: χ^2^ = 65.9239; df = 9; *p* < 0.0001). The EcViR offspring had the highest survival rate, the HA hybrid had the lowest, and the HB, BC1, and BC2 offspring had average survival, regardless of lambda-cyhalothrin dose (Figure 2).

## 4. Discussion

The field-collected population, EcGA, showed a high susceptibility to lambda-cyhalothrin, greater than the population reference for EcFM, which had been reared in the laboratory for 76 generations without insecticide exposure. Furthermore, the field-collected population demonstrated a homogeneous response to lambda-cyhalothrin, requiring close concentrations in the dose response bioassay. It also exhibited a high slope coefficient in comparison to the EcFM and EcViR populations, with a small variation in the 95% confidence limit for LD50 (ca., 0.031 to 0.035). Based on this study and others [16,20], the findings suggest that the susceptibility and resistance of the two EcFM and EcViR populations to lambda-cyhalothrin are at opposite ends of the spectrum. Thus, the findings obtained precisely align with the anticipated outcomes for their offspring. The progeny of the EcFM × EcViR crosses exhibited survival rates ranging from 77.4% to 97.7% when exposed to dried residues of lambda-cyhalothrin at the lowest and highest doses, a trend likewise seen in EcViR individuals. The offspring exhibited a developmental time similar to that of the EcViR and EcFM populations, but it was shorter than that of the parental EcGA individuals. The adults of the EciViR population were of a similar size to those of the EcFM and EcGA populations, and their survival rate was greater than that of the EcFM and EcGA populations.

Additionally, over the 35 days of the adult evaluation period, egg production increased from 33.4% to 54.4% [about 108.7 (HA) to 258.6 (BC1) eggs per female] compared to the EcViR population. This supports the increased fecundity of the F1 offspring produced by crossing EcViR and EcFM [48]. In this way, crosses between EcGA and EcViR have more offspring than EcViR, and they are just as likely to survive treatment with the recommended doses of lambda-cyhalothrin as EcViR. Furthermore, the laboratory-raised EcFM and EcGA populations have adapted to the rearing conditions, exhibiting faster development and larger adults compared to the recently collected field population. The results obtained with the EcViR population, which was also reared for 78 generations under the same laboratory and feeding conditions, corroborate this finding.

The field population’s high susceptibility and homogeneous response may be linked to the operational factor of a lack of selection pressure for pyrethroids at the collection site, as both susceptible and resistant populations of *E. connexa* can be collected in the same region [17], thereby substantiating resistance selection as a microevolutionary process [53]. Populations of *E. connexa* from agroecosystems with extensive insecticide applications, such as cotton and maize, exhibit susceptibility while simultaneously demonstrating resistance levels to lambda-cyhalothrin [17]. Factors like temperature and prey availability promote an increased number of generations and a larger number of individuals, which, in conjunction with the history of insecticide use, intensifies selection pressure for resistance. The resistance of an insect population to an insecticide is influenced by the selection pressure exerted and determined by genetic and physiological characteristics [54,55]. However, distinguishing between the absence and presence of selection pressure in the field for coccinellids is challenging because individuals may disperse across fields and have reduced exposure to insecticide applications. The dual resistance of *H. convergens* to pyrethroids and organophosphates was attributed to the exposure to insecticides applied in cotton, tobacco, walnut, and other agroecosystems within the same region, either concurrently or sequentially [36]. This phenomenon has also been seen in pest species when crop rotation and insecticide modes of action are absent, leading to repeated exposure to the same active ingredient [56].

Regarding the survival of the progenies upon exposure to lambda-cyhalothrin doses, some mortality was observed during the first days of exposure, but not exceeding 15% (Figure 2). Following this interval, the progenies maintained their survival, with the final results ranging from 80.9 to 98.5%. The survival of offspring followed the pattern of a heterozygous phenotype-owning resistance. This occurs when resistant and susceptible parents cross, and the resistance inheritance is autosomal [48,57]. Resistance to lambda-cyhalothrin in the EcViR *E. connexa* after 10 generations of selection is monogenic, but with the possibility of secondary genes exerting impact [44]. Nonetheless, successive generations subjected to selection pressure reduce the variability conferred by these secondary genes [58], which may explain the survival rate up to 98.5% of the offspring, as well as a survival rate of 77.4%, which corresponds with the anticipated survival range for heterozygous individuals from EcGA × EcViR progenies.

Based on the results of the EcGA × EcViR crosses, we can infer this about the progenies: the heterosis effect promotes faster development compared to the EcGA parental population, higher egg production than the EcViR population during the period that likely includes the period of the adults lasting in the field, subjected to variable temperature and prey conditions, and high survival at the recommended doses of lambda-cyhalothrin similar to EcViR. Despite this, it is likely that resistant parents and F1 offspring will mate with susceptible individuals, leading to a generation of resistant offspring. However, further successive crosses with susceptible individuals will likely return the population to a susceptible state [47]. Furthermore, mitigation of pest resistance selection requires the use of different insecticides and modes of action. The EcViR population has high survival rates when exposed to permethrin, cypermethrin, deltamethrin, and fenvarelate, but low survival rates when exposed to fenpropathrin and bifenthrin [35]. Other pyrethroids should also be recommended. In the search for recommendations using different modes of action and pest species, some insecticides have shown no or low acute toxicity to *E. connexa*. These include pymetrozine, chlorantraniliprole, and spinosad [59], spinetoram [60], ethiprole [61], pyriproxyfen, and teflubenzuron [62]. When considering only the adult stage of *E. connexa*, the chitin synthesis inhibitors diflubenzuron, lufenuron, triflumuron, and the pyrethroid beta-cyfluthrin were considered harmless [63]. Cantraniliprole, chlorfenapyr, deltamethrin, and methomyl, applied at the recommended rate in brassica crops in Brazil, resulted in high mortality in turnip aphids; however, they did not affect the survival or predation of *E. connexa* on *P. xylostella* larvae [64].

The release and conservation of natural enemies has been supported in integrated pest control programs. Augmentative biological control includes a variety of parasitoids and predators [65]. These species can be improved for desirable characteristics such as climate tolerance, particularly to high temperatures, the induction or breaking of diapause, and insecticide resistance, among other characteristics [1,66,67]. Issues such as poor environmental adaptability and a susceptibility to insecticides applied to control pest species not targeted by the natural enemy prevent many of these species from achieving the intended results. Again, the fact that certain insecticides exhibit physiological selectivity, along with compatibility arising from resistance selection in natural enemies, may help with their use and conservation. Thus, when deciding to release a natural enemy, the availability of populations of the species with additional characteristics selected to promote their efficiency or conservation after release, such as the resistance shown by *E. connexa*, will optimize the outcomes [43,64].

## 5. Conclusions

Adults from the field-collected (EcGA) population are ~200-fold more susceptible than the resistant population (EcViR). Individuals from the EcGA population exhibited delayed development and smaller adults compared to the EcViR population and their progenies. Despite producing a statistically similar number of eggs, females from the EcGA population and EcGA × EcViR progenies produced 130 more eggs during the 35-day adult evaluation period than the resistant females from the EcViR population. Also, adults from the EcViR × EcGA population had high survival rates when exposed to label doses of lambda-cyhalothrin similar to the resistant EcViR parents. However, adults from the EcGA population and the laboratory standard population for susceptibility (EcFM) had very low or no survival. These findings suggest that field crossings between EcViR and EcGA will improve the biological performance of their progenies compared to their parents, EcViR, and will sustain a high lambda-cyhalothrin survival rate.

## Figures and Tables

**Table 1:** The designed crosses using three *Eriopis connexa* populations: EcGA, EcFM, and EcViR, respectively, at their 1st, 76th, and 78th parental generations in the laboratory, and their respective offspring in the following generations. Note: female (♀) and male (♂) backcross designed according to the autosomal mode of the inherited resistance to lambda-cyhalothrin.

Crossing Designs	Parental Population		Offspring
Reference	♀EcGA	♂EcGA	EcGA
	♀EcFM	♂EcFM	EcFM
	♀EcViR	♂EcViR	EcViR
Reciprocal	♀EcGA	♂EcViR	Hybrid A = HA
	♂EcGA	♀EcViR	Hybrid B = HB
Backcross	HA♀	♂EcViR	Backcross 1 = BC1
	HB♀	♂EcViR	Backcross 2 = BC2

**Table 2:** The relative susceptibility to lambda-cyhalothrin of the three populations (pops) of *Eriopis connexa*, evaluated 48 h after topical treatment with six doses. The chi-square test (χ^2^) was used to check the goodness-of-fit to the Probit model. FL or CI_95_ = 95% fiducial limit intervals, RR = resistance ratio based on LD_50_.

Pops	Gen. ^1^	df	Slope	LD_50_ (FL_95_) ^2^	RR_50_ (CI_95_) ^3^	χ^2^	*p*
EcGA	F2 (195)	4	11.1 ± 1.59	0.036 (0.034–0.037)	-	0.1599	0.99
EcFM	F76 (129)	4	1.1 ± 0.20	0.090 (0.048–0.146)	2.51 (0.75–8.44)	2.0752	0.72
EcViR	F78 (125)	4	3.1 ± 0.54	7.23 (5.82–9.04)	200.71 (165.96–933.28) *	2.5632	0.63

^1^ Current generation when assayed and number of adults treated. ^2^ Estimated doses of lambda-cyhalothrin (mg a.i. mL^−1^) required to kill 50% of treated insects. ^3^ RR calculated through method of Robertson and Preisler [51]; values were judged statistically significant (*), when confidence intervals did not bracket value 1.0 [52].

**Table 3:** Characteristics (means ± SE) obtained from three *Eriopis connexa* populations (EcGA, EcFM, and EcViR) and their reciprocal and backcross offspring.

Crosses (Offspring)	No. of Eggs per Female ^1^	Egg Viability (%)	Larval Duration Time (Days)	Pupation Time (Days)	Immature Survival (%)	Adult Fresh Weight (mg)
♀ × ♂EcGA	267.6 ± 61.7 ab	57.1 ± 6.7 b	20.5 ± 0.4 a	5.2 ± 0.1 c	81.7 ± 5.0 a	7.8 ± 0.3 a
♀ × ♂EcFM	307.8 ± 50.1 a	54.2 ± 4.9 bc	13.3 ± 0.2 b	5.9 ± 0.0 b	95.0 ± 2.8 a	8.2 ± 0.2 a
♀ × ♂EcViR	216.5 ± 47.0 b	61.1 ± 2.2 b	13.6 ± 0.1 b	4.5 ± 0.1 d	88.3 ± 4.2 a	9.2 ± 0.2 a
♀EcGA × ♂EcViR (HA)	325.2 ± 59.3 a	35.4 ± 4.8 c	13.5 ± 0.1 b	4.5 ± 0.01 d	81.7 ± 5.0 a	9.4 ± 0.2 a
♂EcGA × ♀EcViR (HB)	328.5 ± 48.6 a	49.6 ± 2.9 bc	12.7 ± 0.2 b	8.1 ± 0.2 a	81.7 ± 5.0 a	10.0 ± 1.5 a
♀HA × ♂EcViR (BC1)	475.1 ± 43.1 a	84.2 ± 1.3 a	- ^2^	-	-	-
♀HB × ♂EcViR (BC2)	368.9 ± 51.9 a	81.6 ± 2.2 a	-	-	-	-
F	2.27	19.2	120.86	143.74	1.75	1.68
Df	6, 88	6, 86	4, 252	4, 252	4, 295	4, 252
p	0.0437	<0.0001	<0.0001	<0.0001	0.1392	0.1543

^1^ Monitored over a period of 35 days, tallied from onset of oviposition of each female. Analysis by one-way ANOVA. Means followed by the same letter within columns did not differ significantly (Tukey, α = 0.05). ^2^ Characteristics not assessed.

## Data Availability

The datasets and analysis protocols used during the current study are available from the corresponding author on request.
